# Ciliated muconodular papillary tumors of the lung with *KRAS/BRAF/AKT*1 mutation

**DOI:** 10.1186/s13000-017-0651-2

**Published:** 2017-08-22

**Authors:** Emiko Udo, Bungo Furusato, Kazuko Sakai, Leah M Prentice, Tomonori Tanaka, Yuka Kitamura, Tomoshi Tsuchiya, Naoya Yamasaki, Takeshi Nagayasu, Kazuto Nishio, Junya Fukuoka

**Affiliations:** 1 Department of Pathology, Nagasaki University Graduate School of Biomedical Sciences, 1-7-1 Sakamoto, Nagasaki, 852-8501 Japan; 2 Department of Genome Biology, Kindai University Faculty of Medicine, 377-2 Ohno-higashi, Osaka-Sayama, Osaka, 589-8511 Japan; 3 Contextual Genomics, Suite #204 Donald Rix Building 2389 Health Sciences Mall, Vancouver, V6T 1Z3 BC Canada; 4 Department of Surgical Oncology, Nagasaki University Graduate School of Biomedical Sciences, 1-7-1 Sakamoto, Nagasaki, 852-8501 Japan

**Keywords:** Ciliated muconodular papillary tumor, CMPT, Next-generation sequencing, Mutation, *BRAF*, *RAS*, *AKT1*

## Abstract

**Background:**

Ciliated muconodular papillary tumors (CMPTs) are newly recognized rare peripheral lung nodules that are histologically characterized by ciliated columnar, goblet, and basal cells. Although recent studies have shown that CMPTs constitute a neoplastic disease, the complete histogenesis of CMPTs is not fully understood and molecular data are limited.

**Methods:**

We reviewed four cases of CMPT and performed immunohistochemical and genomic analyses to establish CMPT profiles.

**Results:**

All cases were positive for hepatocyte nuclear factor-4α and mucin 5B and negative for programmed death ligand 1 expression, as determined by immunohistochemistry. The genetic analysis revealed three pathogenic mutations (*BRAF* V600E, *AKT1* E17K, and *KRAS* G12D), with the *KRAS* mutation reported here for the first time.

**Conclusion:**

Histological and genetic profiles indicate that CMPTs are likely neoplastic and exhibit features similar to mucinous adenocarcinoma. This suggests that some CMPTs may be a precursor lesion of mucinous adenocarcinoma.

**Electronic supplementary material:**

The online version of this article (doi:10.1186/s13000-017-0651-2) contains supplementary material, which is available to authorized users.

## Background

Ciliated muconodular papillary tumors (CMPTs) are a newly recognized small-size papillary tumor of the peripheral lung that contain columnar cells, occasional basal cells, and mucus-producing cells as well as extracellular mucin pools of various sizes. Although CMPTs were first described based on certain pathological features that suggested a malignant potential, similar diseases such as extremely well-differentiated papillary adenocarcinoma with prominent cilia formation have been reported under different names [1–3]. Due to its complex histology and presence of inflammation and fibrosis, the metaplastic nature of CMPTs has been debated. However, recent reports have revealed the frequent presence of driver gene mutations, and CMPT is now recognized as a neoplasia [4–6]. The histogenesis and molecular characteristics of CMPTs are not well understood owing to the rarity of the disease. To address this issue, we reviewed our case archive and characterized CMPTs by immunohistochemistry and next-generation sequencing (NGS).

## Methods

### Patient selection and tissue preparation

Cases of surgically resected CMPT from 2012 and 2016 were searched in the pathology archive of Nagasaki University Hospital. Hematoxylin and eosin-stained slides of each case were reviewed by two pathologists specializing in thoracic medicine. We identified four CMPT cases; two of these had been originally diagnosed as CMPT, whereas the other cases had been diagnosed as glandular papilloma and mucinous adenocarcinoma. Clinical data were extracted from the hospital’s electronic medical records. Sections with a thickness of 4 μm from formalin-fixed, paraffin-embedded (FFPE) tissue samples were examined by immunohistochemistry, and 15 sections with a thickness of 5 μm were subjected to NGS of 50 cancer-related genes using the Ion Torrent PGM system (Life Technologies, Carlsbad, CA), and10 μm thick section on the conventional glass slide were submitted for 29 genes analysis using MiSeq (Illumina, San Diego, CA, USA).

### Isolation of genomic DNA

Genomic DNA was extracted from FFPE tumor samples using the QIAamp DNA FFPE kit (Qiagen, Valencia, CA, USA), and the concentration was determined using the Quant-iT PicoGreen dsDNA Assay kit (Life Technologies).

### Targeted deep sequencing of mutational hotspots in 50 cancer-related genes

Genomic DNA was subjected to whole-exome sequencing using the Ion AmpliSeq Cancer Hotspot Panel v.2 (Life Technologies) according to the manufacturer’s instructions. The purified library was sequenced on an Ion PGM instrument using Ion PGM Hi-Q Sequencing kit and Ion 318 Chip Kit v.2 (all from Life Technologies). DNA sequencing data were accessed with the Torrent Suite v.5.0 program (Life Technologies). The coverage analysis was performed using the coverage analysis plug-in v5.0. Reads were aligned with the hg19 human reference genome, and potential mutations were identified using Variant Call Format v.5.0. Raw variant calls were annotated with CLC Genomics Workbench software (CLC bio, Aarhus, Denmark). Variants were manually verified using the integrative genomics viewer (Broad Institute, Cambridge, MA, USA). Known single nucleotide polymorphisms were identified using the Human Genetic Variation Database (http://www.hgvd.genome.med.kyoto-u.ac.jp/) [7] and were excluded.

We also performed sequencing on Illumina MiSeq instrument. As recommended by the manufacturer, 50 ng of dsDNA was used for generating sequencing libraries using Find-It cancer hotspot panel (Contextual Genomics, Vancouver, BC, Canada). The panel targets 90+ cancer hotspot mutations including eight coding exons in 29 cancer-related genes. DNA libraries were denatured and diluted as per Illumina’s recommendations. Samples were run on MiSeq machine using 300 cycle MiSeq Reagent Kit V2 (Cat.No.MS-102-2002, Illumina).

### Immunohistochemistry

Immunohistochemical analysis of 4-μm tissue sections was carried out using the Ventana Bench Mark XT Automated stainer (Ventana Medical Systems, Tucson, AZ, USA) and BOND III fully automated stainer (Leica Biosystems, Melbourne, Australia) with antibodies against thyroid transcription factor (TTF)-1 (clone SPT24; Leica Novocastra, Newcastle Upon Tyne, UK), hepatocyte nuclear factor (HNF)-4α (clone H1415; Perseus Proteomics, Tokyo, Japan), mucin (MUC)5B (Santa Cruz Biotechnology, Santa Cruz, CA, USA), MUC5A (clone CLH2; Leica Novocastra), cytokeratin (CK)7 (clone OV-TL12/30; Dako Japan, Tokyo, Japan), CK20 (clone Ks20.8; Dako Japan), p53 (clone DO-7; Leica Novocastra), Ki-67 (clone MIB-1; Dako Japan), Caudal-type homeobox (CDX)2 (clone EPR2764Y; Roche Diagnostics, Indianapolis, IN, USA), ALK (clone D5F3; Roche Diagnostics), BRAF V600E (clone VE1; Roche Diagnostics) and programmed death ligand (PD-L)1 (clone SP142; Spring Bioscience, Pleasanton, CA, USA). Details of optimized staining protocols are listed in Additional file 1. HNF4α, MUC5B, MUC5A, CK7, and CK20 staining was scored as follows: −, negative; 1+, focal; and 2+, diffuse. The intensity of p53 expression was assessed as follows: 1+, faint and sporadic; 2+, frequently positive with no or equivocal overexpression; and 3+, unequivocal and diffuse overexpression. BRAF V600E and ALK expression was judged as positive when strong and diffuse staining was observed in cancer cells. The percentage of Ki-67-immunopositive cells was analyzed using a nuclear algorithm (Nuclear_v.9_v.10.0.0.1798; Imagescope, Leica Biosystems, Nussloch, Germany). PD-L1 expression was scored as the percentage of PD-L1-positive cells among tumor cells. CDX2 expression was scored as follows: −, negative; 1+, ≤ 25% positive; 2+, > 25 and <50% positive; and 3+, ≥ 50% positive. Two observers (E.U. and J.F.) independently scored the staining and a consensus was obtained through discussion when there was a discrepancy.

## Results

### Clinical summary

Clinical data for the four CMPT patients are summarized in Table 1. The patients were all female with a median age of 67 years old. None had a history of smoking. Tumors ranged in size from 8 to 25 mm (median: 11 mm). Lobectomy was performed in three cases, and segmentectomy was carried out in one case.

**Table 1:** Summary of clinical data and detected gene mutations in the past reports and present cases

Author	Age (median)	Sex (M/F)	smoking habit (Y/N)	Driver Mutation	Other Mutations	NGS panel
Kamata et al. [4]	62	7/3	5/5	*BRAF* V600E, 4 cases;﻿ *EGFR* ex19del E746-T751, 3 cases; *BRAF* G606R, 1 case	*IDH1* G123R, 1 case; *CTNNB1* D32N, 1 case; *PTPN11* E76K, 1 case; *PTPN11* P491L, 1 case; *TP53* L289F, 1 case	Ion AmpliSeq Cancer Hotspot Panel v2
Liu et al. [5]	–	1/3	–	*BRAF* V600E, 1 case	*AKT1* E17K, 1 case	Ion AmpliSeq Cancer Hotspot Panel v2
Lau et al. [15]	19	0/1	0/1	none	none	Ion Ampliseq Colon and lung cancer panel
Our series	67	0/4	0/4	*BRAF* V600E 1 case*KRAS* G12D, 1 case	*AKT1* E17K, 1 case	Ion AmpliSeq Cancer Hotspot Panel v2; The Contextual Genomics Find-It^TM^ test

*F* female, *M* male

### Histological findings

**Fig. 1:**
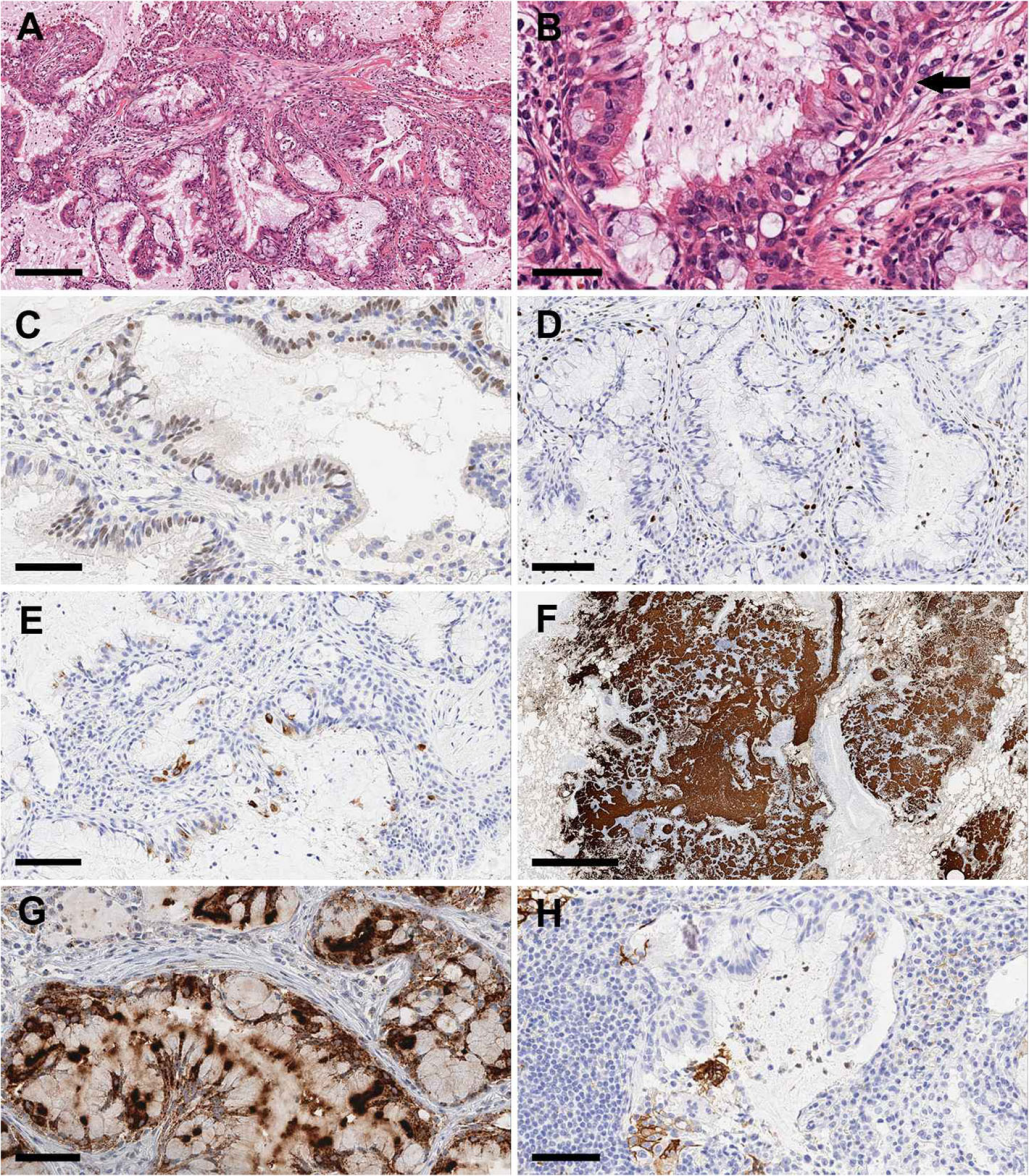
Immunohistochemical analysis of CMPTs**. a** Low magnification view of a CMPT showing papillary epithelial proliferation with abundant mucus production. **b** Higher magnification view of the same case showing a mixture of goblet, ciliated columnar, and basal cells (arrow). **c** HNF-4α positivity in epithelial cell nuclei. Scale bar = 60 μm. **d** The positive rate for Ki-67 expression was <10%. Scale bar = 100 μm. **e** Focal MUC5AC staining in occasional ciliated cells. Scale bar = 90 μm. **f** Epithelial cells and mucin were strongly positive for MUC5B. Scale bar = 2 mm. **g** BRAF V600E staining was strong and diffuse in most epithelial cells of a case harboring *BRAF* V600E and *AKT1* E17K mutations. Scale bar = 60 μm. **h** PD-L1 staining was mostly negative (< 1%); however, focal membranous immunoreactivity was observed. Scale bar = 70 μm

The histological features of the four CMPT cases were consistent with those previously reported [1, 8, 9]. That is, the tumors typically exhibited a mixture of acinar and papillary growth patterns without clear evidence of invasion, and were surrounded by abundant mucus pools in the alveolar spaces (Fig. 1a). The tumors were composed of two basal cell layers and surface epithelia. The latter consisted of an uneven mosaic of ciliated columnar, goblet, and mucin-producing epithelial cells of the gastric type (Fig. 1b). Few tumor cells showed nuclear atypia, and no mitosis or necrosis was observed.

### Immunohistochemistry

All four cases were positive for nuclear HNF4α and TTF-1. Two cases showed diffuse and strong staining for HNF4α, as seen in mucinous adenocarcinoma or colorectal carcinoma (Fig. 1c). The Ki-67 index was low (2.5%–10%), consistent with a less aggressive nature (Fig. 1d). In contrast to mucinous adenocarcinoma, all cases examined in this study showed diffuse TTF-1 positivity and one case showed possible overexpression. There was sparse cytoplasmic expression of MUC5AC (Fig. 1e) while diffuse MUC5B—which is very rare in the normal lung parenchyma—was detected in all cases (Fig. 1f). Nuclear expression of p53 was sporadic in one case and more frequent in two cases, and in one case p53 was clearly overexpressed. There were no or only a few cells that were positive for CDX2 and CK20. p63 expression highlighted the basal layers in all cases, although the area of coverage was focal in two cases and broad in the two others (Table 2).

**Table 2:** Summary of immnohistochemical findings in previouse reports and present cases

Author	TTF-1	Ki67	CK7	CK20	MUC5AC	MUC5B	p53	HNF4α	p63/p40	CDX2	PD-L1	ALK	*BRAF V600E*
Sato et al. [8]	2/2	3%; 10%	2/2	0/2	1/2	-	-	-	-	-	-	-	-
Chuang et al. [14]	1/1	<1%	1/1	0/1	-	-	0/1	-	1/1	-	-	-	-
Chu et al. [13]	1/1	-	1/1	0/1	-	-	-	-	1/1	-	-	-	-
Kon et al. [12]	5/5	<1%; 5	5/5	0/5	0/5	-	0/5	-	5/5	-	-	-	-
Lau et al. [15]	1/1	not increase	1/1	0/1	1/1(weak)	-	-	-	1/1	-	-	-	-
Kamata et al. [4, 11]	+^a^	-	-	-	+^a^ (cliated cells)	-	-	0/10^a^	+^a^	-	-	-	4/4^b^
Ishikawa et al. [16]	3/5	<5% 3; <10% 1	5/5	0/5	-	-	3/3	-	-	-	-	-	-
Taguchi et al. [10]	0/1	3.7%	1/1	0/1	1/1	-	<1%	-	1/1	-	-	1/1	0/1
Jin et al. [6]	1/1	-	1/1	-	-	-	-	-	1/1	-	-	1/1	-
Our series	4/4	<5% 3; <10% 1	4/4	0/4	4/4	4/4	1/4	4/4	4/4^d^	0/4	<1% 4/4	0/4	1/4^c^

-; not available, ^a^numbers of positive/negative not reported, ^b^cases with BRAF mutation, ^c^case with BRAF mutation was positive (1/1), ^d^one case was a few as positive

One case with *BRAF* V600E mutation identified by NGS showed diffuse and strong BRAF V600E staining, while the other three cases showed none (Fig. 1g). There was no *ALK* and *EGFR* mutations identified in our cases. ALK status was also confirmed by immunohistochemistry.

All cases exhibited <1% PD-L1 positivity in tumor cell membranes (Fig. 1h). These findings suggest that CMPTs are tumors that originated from a terminal respiratory unit and are showed features of gastric-type glands.

### Genetic analysis

Gene mutations were detected in two of the four cases (50%) (Table 1). One case harbored *BRAF* V600E and *AKT1* E17K mutations—which was interestingly identical as what have been recently reported [4, 5]—and another had a *KRAS* G12D mutation. The detected mutation status was identical on both Ion PGM instrument system and Illumina MiSeq system. A *KRAS* G12C mutation was found in one of the two other cases; however, the significance of this mutation is unclear due to its low frequency.

## Discussion

We carried out immunohistochemical and molecular analyses of four cases of CMPT and identified a previously unreported *KRAS* mutation in addition to known *BRAF and AKT1* mutations. The findings of *BRAF* and *AKT1* mutations were identical to those reported in recent studies [4, 5]. However, we could not identify either *EGFR* or *ALK* mutation in our cases [4, 6, 10].

Interestingly, the case with a *KRAS* G12D mutation detected by Ion PGM and Illumina method showed fewer basal cell layers, as confirmed by p63 immunostaining, while another case with reduced basal cell layer coverage also had a *KRAS* mutation (G12C). We did not take this mutation into account due to its low frequency by Ion PGM method and negative result by Illumina method. This *KRAS* status could be due to intratumoral heterogeneity—i.e., the few cells harboring this mutation may have been overshadowed by wild-type cells, which constituted the majority of the tumor cells.

Our immunostaining results were consistent with previous reports [6, 8, 10–16]. However, we showed for the first time that CMPTs were positive for HNF-4α and MUC5B. Although these tumors show similarities to mucinous adenocarcinoma, there are significant differences between them such as the presence of cilia in columnar epithelia and basal cell intervention in the latter. In addition, invasive mucinous adenocarcinoma is usually TTF-1-negative and has distinct malignant features [17].

## Conclusion

This is the first report of CMPTs of the lung harboring a *KRAS* mutation. Our findings suggest that CMPT cases are heterogeneous, and some CMPT cases may be a precancerous form of invasive mucinous adenocarcinoma, although additional studies are needed to investigate this possibility.

## Additional file

Additional file 1: Antibodies used for immunohistochemistry. (XLSX 10 kb)

## Abbreviations

(HNF)-4α: Hepatocyte nuclear factor 4α
ALK: Anaplastic lymphoma kinase
CDX2: Caudal-type homeobox 2
CK: Cytokeratin
CMPT: Ciliated muconodular papillary tumor
EGFR: Epidermal growth factor receptor
FFPE: Formalin-fixed, paraffin-embedded
MUC: Mucin
NGS: Next generation sequencing analysis
PD-L1: Programmed death ligand 1
TTF-1: Thyroid transcription factor-1
